# Fluorinated Tetraphosphonate Cavitands

**DOI:** 10.3390/molecules23102670

**Published:** 2018-10-17

**Authors:** Alessandro Pedrini, Federico Bertani, Enrico Dalcanale

**Affiliations:** Department of Chemistry, Life Sciences and Environmental Sustainability, University of Parma, Parco Area delle Scienze 17/A, 43124 Parma, Italy; alessandro.pedrini@studenti.unipr.it (A.P.); federico.bertani@studenti.unipr.it (F.B.)

**Keywords:** phosphonate cavitands, fluorine, molecular recognition, sarcosine methyl ester, molecular probe

## Abstract

Two synthetic protocols for the introduction of fluorine atoms into resorcinarene-based cavitands, at the lower and upper rim, respectively, are reported. Cavitand **1**, bearing four fluorocarbon tails, and cavitand **2**, which presents a fluorine atom on the *para* position of a diester phosphonate phenyl substituent, were synthesized and their complexation abilities toward the model guest sarcosine methyl ester hydrochloride were evaluated via NMR titration experiments. The effect of complexation on the ^19^F NMR resonance of the probe is evident only in the case of cavitand **2**, where the inset of the cation-dipole and H-bonding interactions between the P=O bridges and the guest is reflected in a sizable downfield shift of the fluorine probe.

## 1. Introduction

Cavitands [1] are programmable abiotic receptors capable of hosting shape-complementary guests through specific weak interactions, such as hydrogen bonding, π-π stacking, CH-π and cation-π interactions. Their remarkable and versatile molecular recognition properties have been exploited in many different fields, including catalysis [2,3,4,5], crystal engineering [6], molecular grippers [7], protein recognition [8,9], responsive nanostructures [10,11], self-diagnostic polymers [12] and sensing [13,14].

Expanding further the application fields of cavitands requires the exploration of new synthetic pathways for the introduction of specific reporting units. A potential but yet unexplored probe is fluorine, with this nucleus being exceptionally sensitive toward molecular and micro-environmental changes [15]. The lack of background interference makes fluorine-19 nuclear magnetic resonance (^19^F NMR) spectroscopy an ideal tool for the study of complex matrices, in particular for in vivo applications, where an endogenous signal from tissues is almost absent.

^19^F NMR spectroscopy was first applied to the study of the complexation properties of calix[4]arene-based molecular receptors by Swager and co-workers in 2013 [16]. Tungsten calix[4]arene imido complexes were decorated with a fluorine atom to unambiguously detect targeted neutral organic molecules. In their design, the perturbation of the electron density generated by the binding of a Lewis basic molecule on tungsten induces a variation of the chemical shift of the π-conjugated fluorine, with a response that highly depends on the electron donating ability of the analyte. The discrimination between different analytes was possible only when the interactions with the tungsten were strong enough to produce static structures on the NMR time scale and peaks at precise chemical shifts. The same group further implemented this approach both by developing an array of fluorinated receptors and by incorporating multiple nonequivalent fluorine atoms into a single receptor [17].

Fluorinated chiral palladium pincer complexes, instead, were successfully applied to an ^19^F NMR-assisted simple and precise differentiation of chiral amines [18]. Diastereomeric complexes resulting from the bonding of enantiomers present a distinct and precise ^19^F NMR fingerprint even in the presence of structurally similar analytes, allowing the simultaneous identification of multiple species. The robustness of this chemosensory platform was demonstrated by the quantification of caffeine content in coffee as well as by the identification of other ingredients in beverages [19].

More recently, Mancin et al. coated gold nanoparticles with fluorinated phenylboronic acids and thioundecyl-d-glucopyranosides. The resulting chemosensor is able to detect dopamine in human urine at physiologically relevant concentrations by means of ^19^F spectroscopic analysis via displacement assay [20].

**Chart 1:**
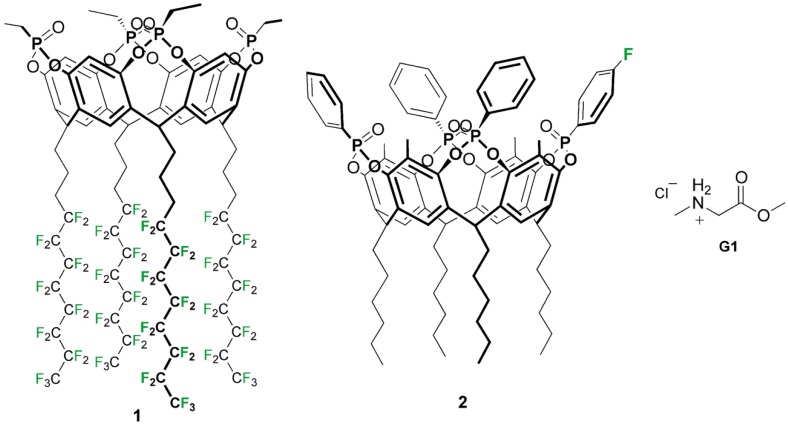
Structure of lower and upper rim fluorinated tetraphosphonate cavitands (**1** and **2** respectively) and sarcosine methyl ester hydrochloride (**G1**).

In view of their applications in biosensing [21], we selected the tetraphosphonate cavitands (Tiiii) [13] as the most valuable receptors for the insertion of fluorine probes. Herein, we report two synthetic protocols for the introduction of fluorine atoms into diester phosphonates [22,23] bridged cavitand scaffolds at the lower and upper rim, respectively (Chart 1) and the response of the fluorine probes toward the complexation of sarcosine methyl ester hydrochloride, as a proxy of sarcosine. The early-stage detection of aggressive prostate cancer has been linked to the presence of sarcosine in urine [24]. In detail, cavitand **1** bearing four fluorocarbon tails is designed to be embedded in fluorine containing nanoemulsions to impart molecular recognition properties [25], while cavitand **2,** presenting a single fluorine atom on one phosphonate bridge, is considered a model system for molecular recognition via NMR using gold nanoparticles [20].

## 2. Results and Discussion

### 2.1. Synthesis of Cavitand **1**

**Scheme 1:**
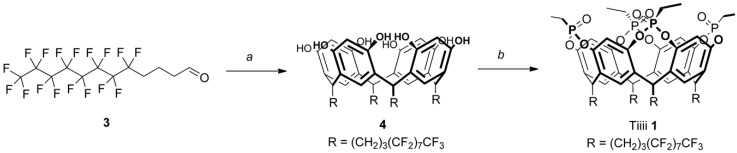
Synthesis of fluorinated tetraphosphonate cavitand **1**: (***a***) resorcinol, HCl 37%, EtOH, 80 °C, 4 h, 23%; (***b***) (1) EtPCl_2_, pyridine/α,α,α-trifluorotoluene, 80 °C, 3 h; (2) H_2_O_2_, r.t, 1 h, 23% (over two steps).

Fluorocarbon-footed tetraphosphonate cavitand **1** was prepared from resorcinarene **4**, bearing four perfluorooctyl moieties. Following similar procedures to those reported in the literature [26,27], the synthesis of resorcinarene **4** required the acidic condensation of resorcinol and a fluorinated aldehyde. Aldehyde **3** was obtained via oxidation with Dess–Martin periodinane of the corresponding perfluorinated alcohol [28], which was obtained in two steps starting from heptadecafluoro-1-iodooctane [29]. The presence of three methylene groups mitigates the electron withdrawing effect of fluorides on aldehyde reactivity. Aldehyde **3** was reacted with an equimolar amount of resorcinol in acidic conditions, affording resorcinarene **4** (Scheme 1). Reaction with dichloroethylphosphine and the subsequent in situ oxidization with hydrogen peroxide afforded diester phosphonate-bridged cavitand **1**, with all P=O groups pointing inward towards the cavity (hence the denomination Tiiii, where *i* stands for *inward*), which was characterized by ^1^H, ^19^F, ^31^P NMR spectroscopy (Figures S1–S3) and matrix-assisted laser desorption ionization time-of-flight (MALDI-TOF) spectrometry (Figures S7 and S8). As expected by the presence of a relevant non-fluorinated part of the molecule, cavitand **1** was found to be insoluble in perfluorohexane, but it presented a good solubility in fluorous hybrid solvents such as methoxyperfluorobutane (HFE-7100).

### 2.2. Synthesis of Cavitand **2**

**Scheme 2:**
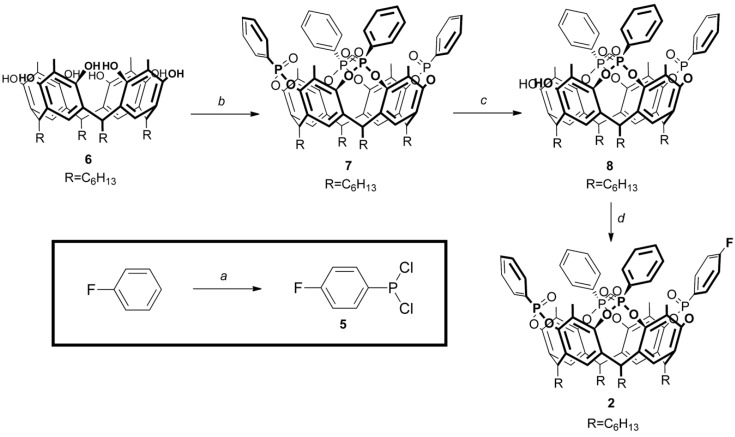
Synthesis of fluorinated tetraphosphonate cavitand **2**: (***a***) (1) PCl_3_, AlCl_3_, 75 °C, 4 h; (2) POCl_3_, r.t., 0.5 h; (***b***) (1) PhPCl_2_, pyridine, 80 °C, 4 h; (2) H_2_O_2_, r.t, 0.5 h, 40% (over two steps); (***c***) 1 eq. 1,2-dihydroxybenzene, K_2_CO_3_, DMF, 80 °C, 5 h, 56%; (***d***) (1) **5**, pyridine, 100 °C, 2 h; (2) H_2_O_2_, r.t, 0.5 h, 10% (over two steps).

Asymmetric tetraphosphonate cavitand **2** was prepared following a convergent synthetic strategy (Scheme 2). In the first step, dichloro(4-fluorophenyl)phosphine (**5**) was synthesized from fluorobenzene via Friedel–Crafts reaction with phosphorous trichloride, using aluminum chloride as a Lewis acid [30]. The subsequent addition of phosphorous oxychloride allowed the removal of AlCl_3_, that precipitated as AlCl_3_·POCl_3_ complex [31]. Compound **5** was characterized via ^1^H, ^19^F and ^31^P NMR; in particular, the presence of the phosphorous signal at 158.7 ppm confirmed the isolation of the substituted phosphorous(III) dichloride species [30]. For its high reactivity towards oxidation and hydrolysis, compound **5** was maintained in solution under an inert atmosphere and used without further purification in the next step. In principle, cavitand **2** could be obtained by the bridging of the corresponding resorcinarene scaffold with a mixture of phosphine **5** and unfunctionalized dichlorophenylphosphine. In our experience, the isolation of the desired product from the mixture of statistical products is difficult [32], and therefore the bridging/excision protocol is to be preferred. Following these considerations, pristine tetraphosphonate cavitand **7** was prepared from tetrahexyl-footed resorcinarene **6** following previously reported procedures [33,34]. The bridging reaction with dichloroethylphosphine in pyridine gave P(III) intermediate, which was oxidized in situ with hydrogen peroxide to afford cavitands **7** with the four diester phosphonate groups directed towards the cavity. Adapting a published protocol [35], one of the four P=O bridges was selectively removed using a stoichiometric amount of catechol as a scavenger and K_2_CO_3_ as a base, affording triphosphonate cavitand diol (**8**) in good yield. In the final reaction, a mixture of **8** and phosphine **5** in pyridine was initially heated at 100 °C for four hours to obtain a mixed-valence phosphorous intermediate that was oxidized in situ to restore the diester phosphonate-decorated rim with the four P=O directed inside the cavity. Fluorine-decorated tetraphosphonate cavitand **2** was isolated in low yield (10%) after column chromatography, as a consequence of the high polarity of the product which hampers the purification step, and completely characterized via NMR spectroscopy (Figures S4–S6) and MALDI-TOF spectrometry (Figures S9 and S10). As expected by the desymmetrization of the cavity, ^31^P NMR spectrum of **2** presented three singlets at 4.4, 7.8 and 8.9 ppm, respectively, with a 1:2:1 ratio of the integrals, while only one fluorine signal at 100.45 ppm was detected with ^19^F NMR technique (Figure S6).

### 2.3. NMR Titration Experiments

The effective variation of the ^19^F NMR chemical shift was evaluated upon complexation with a well-studied guest, namely sarcosine methyl ester hydrochloride (**G1**), for both cavitands [36]. The design of cavitand **2** requires the decoration of a diester phosphonate phenyl substituent with fluorine in *para* position to ensure its π-conjugation with the complexation active P=O group, directly involved in the complexation via cation-dipole and H-bonding interactions. Cavitand **1** instead is designed to probe the complexation by sensing the presence of the chloride counterion, rather than directly sensing the complexation of the protonated sarcosine **G1**. The typical positioning of the chloride counterions in the complexation of sarcosine and related amino acids is between the alkyl feet both in solution and in the solid state to minimize the ion pair distance [36].

**Figure 1:**
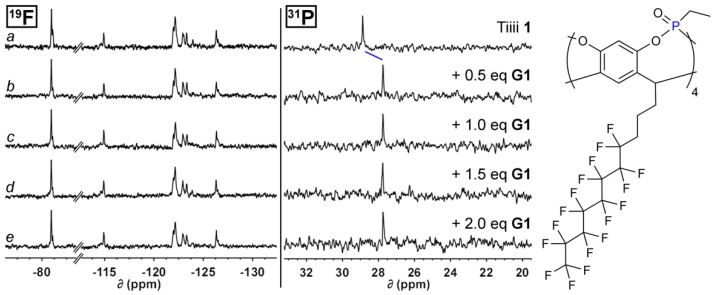
^19^F (376 MHz, CDCl_3_, 298 K) and ^31^P NMR (162 MHz, CDCl_3_, 298 K) spectra of free host **1** (***a***), **1** + 0.5 eq. of **G1** (***b***), **1** + 1.0 eq. of **G1** (***c***), **1** + 1.5 eq. of **G1** (***d***), **1** + 2.0 eq. of **G1** (***e***); on the right cavitand **1** structure is reported.

To a solution of Tiiii **1** or **2** in deuterated chloroform at room temperature, four aliquots of a solution of **G1** in the same solvent were added to reach a guest/host molar ratio of 2. For each addition, ^1^H, ^19^F and ^31^P NMR spectra were recorded and chemical shift variations for both host and guest signals were analyzed. At the initial working concentration, cavitand **1** signals in both proton (Figure S11a) and fluorine (Figure 1a) spectra are broadened. This behavior can be explained by the low solubility of the receptor in the non-fluorinated solvent, which can give rise to the formation of aggregates. Interestingly, upon the addition of the first aliquot of **G1**, a sensible sharpening of the signals in the two spectra was observed, together with an upfield shift of the P=O singlet in ^31^P NMR spectrum (Figure S11b and Figure 1b, right). This is an unprecedented observation, as usually complexation lowers the electron density on the phosphorous atom causing a downfield shift [37]. The reason for this unusual behavior can be traced in the ^31^P chemical shift of free cavitand **1**, which is unusually downfield-shifted with respect to the non-fluorinated analogues in CDCl_3_ (Figure S16), while in the complexed form, the ^31^P chemical shift remains the same both for fluorinated and non-fluorinated cavitands (27.7 ppm compared to 27.4 ppm).

Except for the line shape modification mentioned above, fluorocarbon-tail ^19^F NMR signals do not experience any significant perturbation (Figure 1b, left). Guest proton signals, instead, present upfield shits typical of *N*-methyl ammonium guest complexation in the electron rich cavity. The NCH_3_ triplet, in particular, experienced the highest complexation-induced shifts (from 2.38 to −1.11 ppm), as expected by the deeper inclusion of this group in the cavity. Further additions of **G1** (Figure S11c–e and Figure 1c–e) did not alter significantly the position of both the host and the included guest signals for all the studied nuclei, indicating a saturation of the receptor sites as consequence of the reduced concentration of the free host in solution caused by the presence of aggregates.

**Figure 2:**
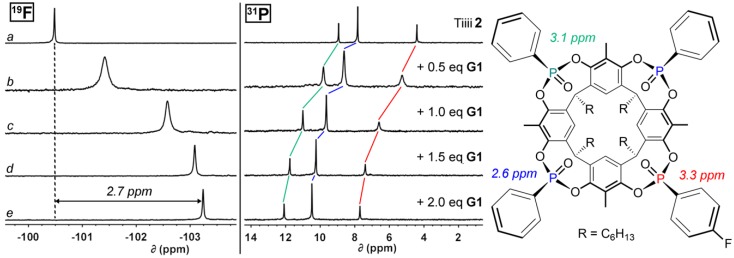
^19^F (376 MHz, CDCl_3_, 298 K) and ^31^P NMR (162 MHz, CDCl_3_, 298 K) spectra of free host **2** (***a***), **2** + 0.5 eq. of **G1** (***b***), **2** + 1.0 eq. of **G1** (***c***), **2** + 1.5 eq. of **G1** (***d***), **2** + 2.0 eq. of **G1** (***e***); on the right chemical shift variations for the three phosphorus signals are reported.

The NMR titration experiment with cavitand **2** and **G1** was performed following the same procedure. The ^1^H NMR spectra (Figures S12–S15) acquired during the titration shows reduced but detectable upfield shifts of the guest NCH_3_ group approaching 1 eq. of **G1**, with a reverse trend and a signal broadening for further additions. This behavior is an indication of a fast exchange regime. In ^19^F NMR spectra (Figure 2a–e, left) a 2.7 ppm upfield shift of the fluorine singlet (from −100.5 to −103.2 ppm) was detected. This result confirmed the effect of the π-conjugation of the fluorine atom with the P=O group through the aromatic ring. For the three singlets in ^31^P NMR spectra (Figure 2a–e, right) different upfield shifts were observed, with appreciable higher values for the P=O group bearing the fluorine-functionalized phenyl substituent and the one in the opposite position (red and green lines in Figure 2).

## 3. Experimental

### 3.1. Materials and Methods

Unless stated otherwise, reactions were conducted in flame-dried glassware under an atmosphere of argon using anhydrous solvents (either freshly distilled or passed through activated alumina columns). All commercially obtained reagents were used as received unless otherwise specified. Silica column chromatography was performed using silica gel 60 (Fluka 230–400 mesh or Merck 70–230 mesh). NMR spectra were obtained using a Bruker AVANCE 400 (400 MHz) spectrometer at 298 K. ^1^H NMR chemical shifts (δ) were reported in ppm relative to the proton resonances resulting from incomplete deuteration of the NMR solvents. ^19^F NMR chemical shifts (δ) were reported in ppm relative to external CF_3_COOH. ^31^P NMR chemical shifts (δ) were reported in ppm relative to external 85% H_3_PO_4_. High-resolution MALDI-TOF was performed on an AB SCIEX MALDI TOF-TOF 4800 Plus (matrix: α-cyano-4-hydroxycinnamic acid). High resolution ESI-LTQ Orbitrap MS analyses were performed with a Linear Trap Quadrupole-Orbitrap mass spectrometer. 1H,1H,2H,2H,3H,3H-heptadecafluorododecanal (**3**) [28] and resorcinarene **6** [38] were prepared according to methods in the literature. The adopted nomenclature follows an established rule [13].

### 3.2. Syntheses

#### 3.2.1. Synthesis of Resorcinarene [(CH_2_)_3_(CF_2_)_7_CF_3_, H] (**4**)

To a solution of resorcinol (0.112 g, 1 mmol) in 2 mL of EtOH, a 37% solution of HCl (0.3 mL, 4 mmol) was slowly added at 0 °C. At the same temperature, a solution of aldehyde **3** (0.500 g, 1 mmol) in 3 mL of EtOH was added dropwise. The mixture was allowed to warm over 90 min and then heated at 80 °C for 4 h. After cooling, the solvent was removed and the crude was purified by flash column chromatography (hexane/EtOAc 1:3). Resorcinarene **4** was obtained as a white solid (0.136 g, 0.06 mmol, 23%).

^1^H NMR (CD_3_OD, 400 MHz): δ (ppm) = 7.19 (s, 4H, ArH_up_), 6.27 (s, 4H, ArH_down_), 4.37 (t, 4H, *J* = 7.8 Hz, CH), 2.31–2.16 (m, 16H, CHC*H*_2_ + C*H*_2_CF_2_), 1.61 (m, 8H, CH_2_); ^19^F NMR (CD_3_OD, 376 MHz): δ (ppm) = −82.6 (t, 3F, *J*_F-F_ = 10 Hz, CF_3_), −115.6 (t, 2F, *J*_F-F_ = 15 Hz, CF_2_CH_2_), −122.8 (m, 2F), −123.1 (m, 4F), −123.9 (m, 2F), −124.8 (m, 2F), −127.5 (m, 2F); ESI-FT-Orbitrap-MS: calculated for C_72_H_43_F_68_O_8_ [M − H]^−^ *m/z* = 2327.187, found *m/z* = 2327.181.

#### 3.2.2. Synthesis of Tiiii [(CH_2_)_3_(CF_2_)_7_CF_3_, H, Et] (**1**)

Resorcinarene **4** (0.060 g, 0.025 mmol) was suspended in 5 mL of a 2:1 mixture of pyridine/α,α,α-trifluorotoluene and dichloroethylphosphine (0.012 mL, 0.11 mmol) was added. The mixture was heated at 80 °C for 3 h. After cooling, 0.5 mL of aqueous 35% H_2_O_2_ was added at 0 °C and the mixture was stirred for 1 h. The reaction was quenched with 20 mL of water and extracted with a 1:1 mixture of CHCl_3_/HFE-7100. The organics were washed with water and the solvent was removed under reduced pressure. Cavitand **1** (0.015 g, 0.006 mmol, 23%) was obtained as a white solid.

^1^H NMR (CDCl_3_, 400 MHz): δ (ppm) = 8.88 (s, 4H, ArH_up_), 6.59 (s, 4H, ArH_down_), 4.50 (t, 4H, *J* = 8.0 Hz, CH), 3.11 (m, 8H, CHC*H*_2_), 2.36 (m, 8H, C*H*_2_CF_2_), 2.21 (m, 8H, P(O)C*H*_2_CH_3_), 1.62 (m, 8H, CHCH_2_C*H*_2_), 1.43 (m, 12H, P(O)CH_2_C*H*_3_); ^19^F NMR (CD_3_OD, 376 MHz): δ (ppm) = −80.9 (t, 3F, *J*_F-F_ = 10 Hz, CF_3_), −115.0 (m, 2F, CF_2_CH_2_), −122.0 (m, 2F), −122.2 (m, 4F), −123.0 (m, 2F), −123.3 (m, 2F), −126.3 (m, 2F); ^31^P NMR (CDCl_3_, 162 MHz): δ (ppm) = 28.9 (s, 4P); MALDI-TOF: calculated for C_80_H_57_F_68_O_12_P_4_ [M + H]^+^ *m*/*z* = 2625.171, found *m*/*z* = 2625.125; calculated for C_80_H_56_F_68_O_12_P_4_Na [M + Na]^+^ *m*/*z* = 2647.153, found *m*/*z* = 2647.135; calculated for C_80_H_56_F_68_O_12_P_4_K [M + K]^+^ *m*/*z* = 2663.127, found *m*/*z* = 2663.124.

#### 3.2.3. Synthesis of Dichloro(4-fluorophenyl)phosphine (**5**)

A mixture of fluorobenzene (1.0 mL, 10.6 mmol), PCl_3_ (3.72 mL, 42.6 mmol) and AlCl_3_ (1.85 g, 13.8 mmol) was heated at 75 °C for 4 h. POCl_3_ (1.3 mL, 13.8 mmol) was carefully added to the hot reaction mixture and the precipitate was filtered under an inert atmosphere and washed with diethyl ether. Removal of the solvent under reduced pressure afforded phosphine **5**, that was used without purification for the next step.

^1^H NMR (CDCl_3_, 400 MHz): δ (ppm) = 7.93 (m, 2H, ArH_3,5_), 7.23 (t, 2H, *J* = 7.8 Hz, ArH_2,6_); ^19^F NMR (CDCl_3_, 376 MHz): δ (ppm) = −105.2 (s, 1F); ^31^P NMR (CDCl_3_, 162 MHz): δ (ppm) = 158.7 (s, 1P).

#### 3.2.4. Synthesis of Tiiii [C_6_H_13_, CH_3_, Ph] (**7**)

Dichlorophenylphosphine (0.693 mL, 5.11 mmol) was added slowly at room temperature to a solution of resorcinarene **6** (1.0 g, 1.14 mmol) in freshly distilled pyridine (30 mL). The mixture was stirred at 80 °C for 4 h. After cooling to room temperature, aqueous 35% H_2_O_2_ (2 mL) was added and the resulting mixture was stirred for 30 min at room temperature. H_2_O (200 mL) was added and the precipitate was filtered, washed with water and dried. The crude was purified by flash column chromatography (silica gel, CH_2_Cl_2_/EtOH 9:1) affording cavitand **7** as a white solid (0.622 g, 0.45 mmol, 40%).

^1^H NMR (CDCl_3_, 400 MHz): δ (ppm) = 8.15 (m, 8H, ArH_o_), 7.67 (m, 4H, ArH_p_), 7.57 (m, 8H, ArH_m_), 7.17 (s, 4H, ArH_down_), 4.82 (t, 4H, *J* = 7.9 Hz, CH), 2.37–2.28 (m, 20H, CHC*H*_2_ + ArCH_3_), 1.54–1.35 (m, 32H, CH_2_), 0.94 (t, 12H, *J* = 6.7 Hz, CH_2_C*H*_3_); ^31^P NMR (CDCl_3_, 162 MHz): δ (ppm) = 6.3 (s, 4P).

#### 3.2.5. Synthesis of Cavitand 3POiii [C_6_H_13_, CH_3_, Ph] (**8**)

Catechol (0.036 g, 0.33 mmol) and K_2_CO_3_ (0.454 g, 3.28 mmol) were added to a solution of cavitand **7** (0.45 g, 0.33 mmol) in 20 mL of DMF. The mixture was heated at 80 °C under stirring for 4 h. After cooling at room temperature, the solvent was removed under reduced pressure and the residue was recovered with CH_2_Cl_2,_ washed with 1N aqueous solution of HCl, water and brine. The organic layer was dried over Na_2_SO_4_ and evaporated to dryness. The crude product was purified by flash column chromatography (silica gel, CH_2_Cl_2_/EtOH 95:5) to give cavitand **8** as an off-white solid (0.210 g, 0.19 mmol, 56%).

^1^H NMR (CDCl_3_, 400 MHz): δ (ppm) = 8.11 (m, 6H, ArH_o_), 7.69 (m, 3H, ArH_p_), 7.59 (m, 6H, ArH_m_), 7.23 (s, 2H, ArH_down_), 7.10 (s, 2H, ArH_down_), 4.74 (m, 3H, CH), 4.46 (m, 1H, CH), 2.32 (m, 6H, CHC*H*_2_), 2.19 (m, 14H, ArCH_3_ + CHC*H*_2_), 1.52–1.30 (m, 32H, CH_2_), 0.93 (m, 12H, CH_2_C*H*_3_); ^31^P NMR (CDCl_3_, 162 MHz): δ (ppm) = 8.4 (s, 2P), 7.9 (s, 1P).

#### 3.2.6. Synthesis of Tiiii [C_6_H_13_, CH_3_, 3Ph + 1PhF_p_] (**2**)

Cavitand **8** (0.200 g, 0.18 mmol) was dissolved in 10 mL of pyridine and phosphine **5** (0.18 mL, 1M solution in diethyl ether) was added. The mixture was heated at 100 °C for 2 h under stirring. After cooling to room temperature, aqueous 35% H_2_O_2_ (1 mL) was added and the resulting mixture was stirred for 30 min at room temperature. H_2_O (100 mL) was added and the precipitate was filtered, washed with water and dried. The crude mixture was purified by flash column chromatography (silica gel, CH_2_Cl_2_/EtOH 9:1) affording cavitand **2** as a white solid (0.016 g, 0.02 mmol, 10%).

^1^H NMR (CDCl_3_, 400 MHz, see supplementary material for signal attribution): δ (ppm) = 8.13 (m, 2H, H_a’’_), 7.98 (m, 4H, H_a’_), 7.72–7.46 (m, 9H, H_b’’_ + H_b’_ + H_c’_+ H_c’’_), 7.27 (s, 2H, H_down’_), 7.25 (s, 2H, H_down_), 6.44 (m, 4H, H_a_ + H_b_), 4.91 (t, 1H, *J* = 6.6 Hz, H_d’’_) 4.77 (m, 3H, H_d’_ + H_d_), 2.35 (m, 8H, CHC*H*_2_), 2.20 (s, 6H, H_d’_), 1.66 (s, 6H, H_d’_), 1.57–1.24 (m, 32H, CH_2_), 0.91 (t, 12H, *J* = 6.3 Hz, CH_3_); ^19^F NMR (CDCl_3_, 376 MHz): δ (ppm) = −100.5 (s, 1F); ^31^P NMR (CDCl_3_, 162 MHz): δ (ppm) = 8.9 (s, 1P), 7.8 (s, 2P), 4.5 (s, 1P). MALDI-TOF: calculated for C_80_H_92_FO_12_P_4_ [M + H]^+^ *m*/*z* = 1387.552, found *m*/*z* = 1387.546; calculated for C_80_H_91_FO_12_P_4_Na [M + Na]^+^ *m*/*z* = 1409.534, found *m*/*z* = 1409.521.

## 4. Conclusions

The present study describes two possible synthetic ways to introduce fluorine probes in tetraphosphonate cavitands, at the lower and upper rim, respectively. In the first case, several fluorine groups have been introduced as the final portion of the four alkyl feet of the cavitand **1**, where the key step is the synthesis of fluorinated resorcinarene **4**. In the second case, a single fluorine group has been introduced on the phenyl substituent of one P=O bridge to give cavitand **2**. The preferred synthetic approach turned out to be the excision of tetraphosphonate cavitand **7** to give the tri-bridged intermediate **8**, which is then bridged with the phosphine **5** and oxidized to give the desired cavitand **2** with all four P=O pointing toward the cavity.

The perturbation of the fluorine probe upon complexation was assessed in both cases using sarcosine methyl ester hydrochloride (**G1**) as a prototype guest. The effect of complexation on the ^19^F NMR resonance of the probe is evident only in the case of cavitand **2**, where the inset of cation-dipole and H-bonding interactions between the P=O bridges and the guest is reflected in a sizable downfield shift of the fluorine probe. The same complexation does not perturb the fluorine probes at the lower rim of cavitand **1**. The nesting of the chloride counterion between the alkyl feet in the complex [34] does not affect the fluorine probes’ chemical shift, possibly because they are remote with respect to the chloride position. The alternative of fully fluorinated alkyl feet is synthetically not viable, due to the reduced reactivity of perfluorinated aldehydes. Therefore, the positioning of the fluorine probes on the P=O bridges is the best option for potential biological applications.

## Supplementary Material

NMR spectra of cavitands **1** and **2** (Figures S1–S6), MALDI-TOF spectra of cavitands **1** and **2** (Figures S7–S10), ^1^H NMR titration spectra for complexation of cavitand **1** and **2** with **G1** (Figures S11–S16).
