# Supplementary material for: Fluorinated Tetraphosphonate Cavitands
Source: Molecules. 2018 Oct 17;23(10):2670. doi: 10.3390/molecules23102670 (PMC6222714; doi:10.3390/molecules23102670)
Supplement: Supplementary file 1 [file molecules-23-02670-s001.docx]

**Supplementary material for**

**Fluorinated tetraphosphonate cavitands**

Alessandro Pedrini,^a^ Federico Bertani,^a^ Enrico Dalcanale^a,*^

a. Department of Chemistry, Life Science and Environmental Sustainability, University of Parma, Parco Area delle Scienze 17/A, 43124 Parma, Italy; E-Mails: alessandro.pedrini@studenti.unipr.it (A. P.); Federico.bertani@intercos.it (F. B.); enrico.dalcanale@unipr.it (E. D.).

***** Author to whom correspondence should be addressed; E-Mail: [enrico.dalcanale@unipr.it](mailto:enrico.dalcanale@unipr.it); Tel.: +39-0521-905463.

**Summary**

[1 Cavitands characterization S2](#_Toc525862421)

[1.1 NMR Spectra S2](#_Toc525862422)

[1.2 Mass spectra S5](#_Toc525862423)

[2 NMR titration experiments S7](#_Toc525862424)

# Cavitands characterization

## NMR Spectra


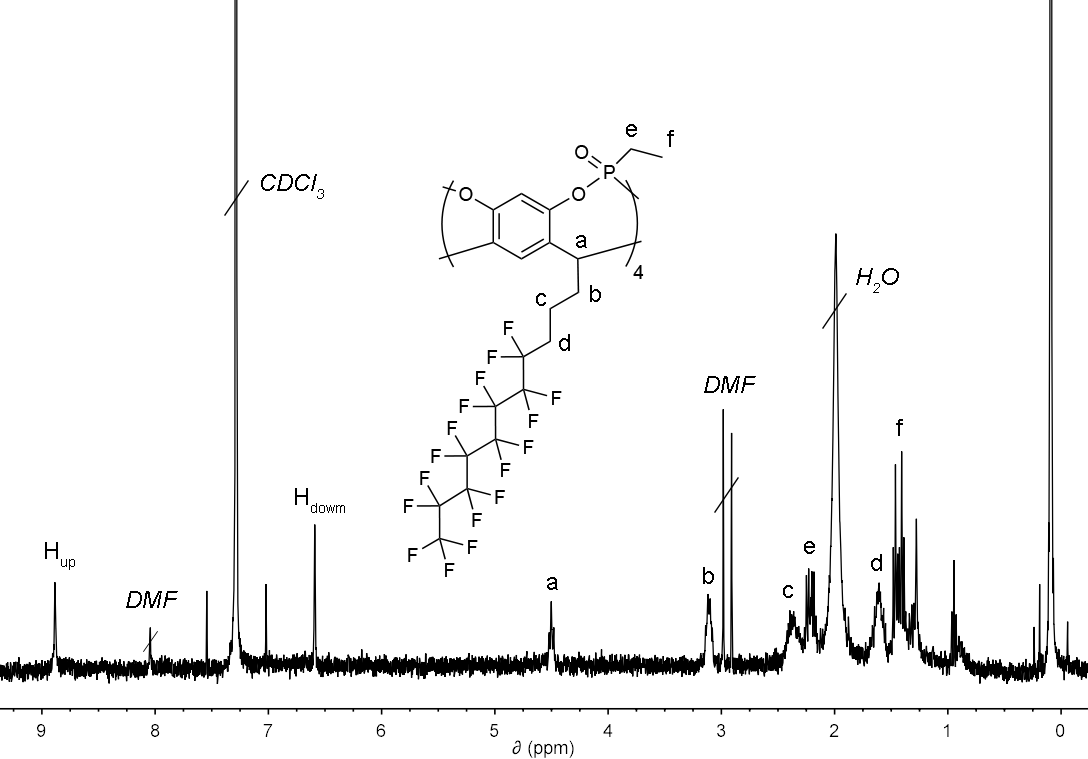


**Figure S1.** ^1^H NMR spectrum (CDCl_3_, 400 MHz, 298 K) of Tiiii [(CH_2_)_3_(CF_2_)_7_CF_3_, H, Et] (**1**)


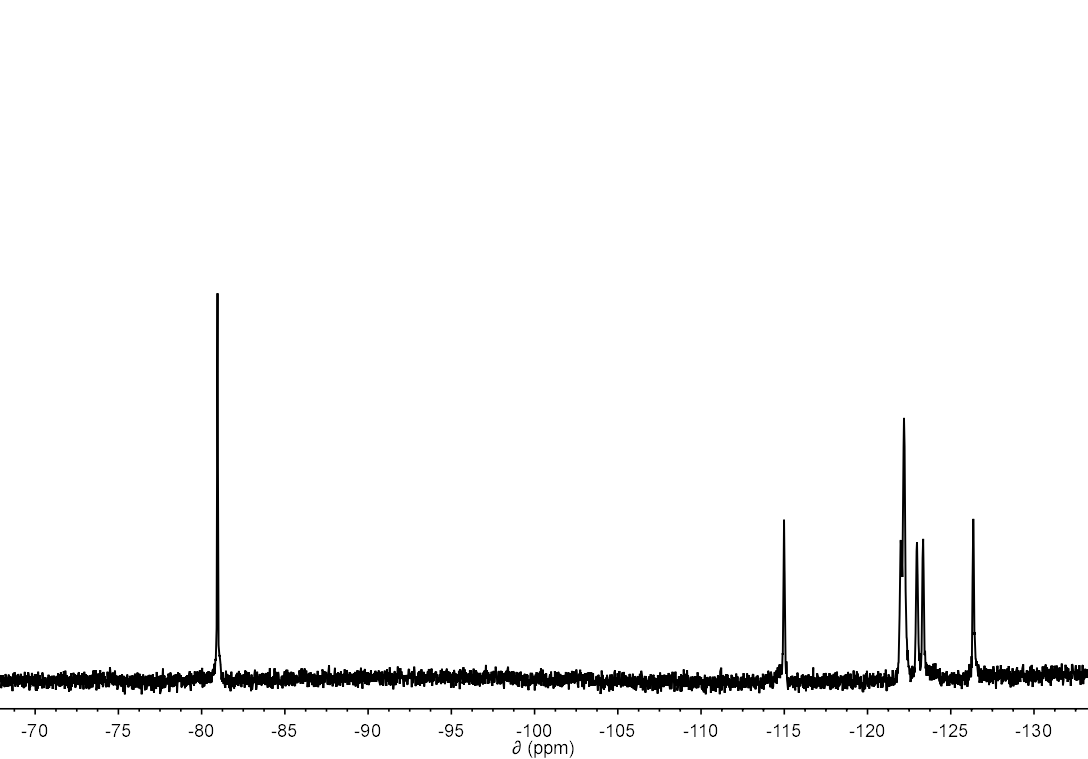


**Figure S2.** ^19^F NMR spectrum (CDCl_3_, 376 MHz, 298 K) of Tiiii [(CH_2_)_3_(CF_2_)_7_CF_3_, H, Et] (**1**)


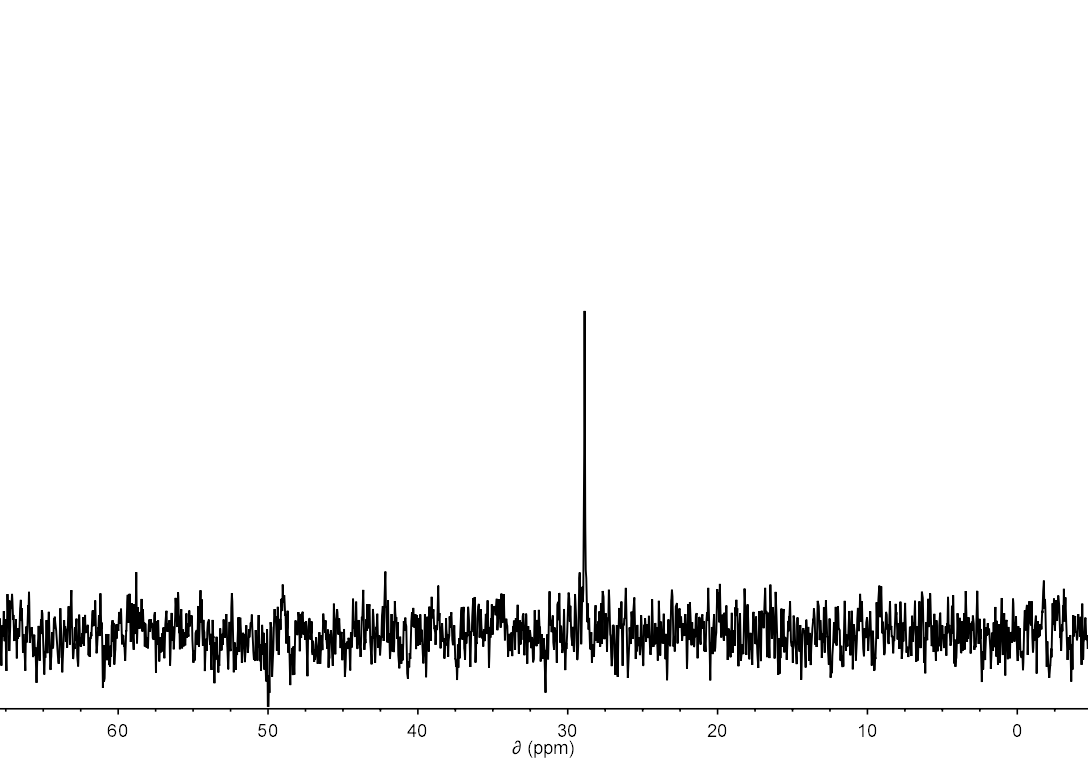


**Figure S3.** ^31^P NMR spectrum (CDCl_3_, 162 MHz, 298 K) of Tiiii [(CH_2_)_3_(CF_2_)_7_CF_3_, H, Et] (**1**)


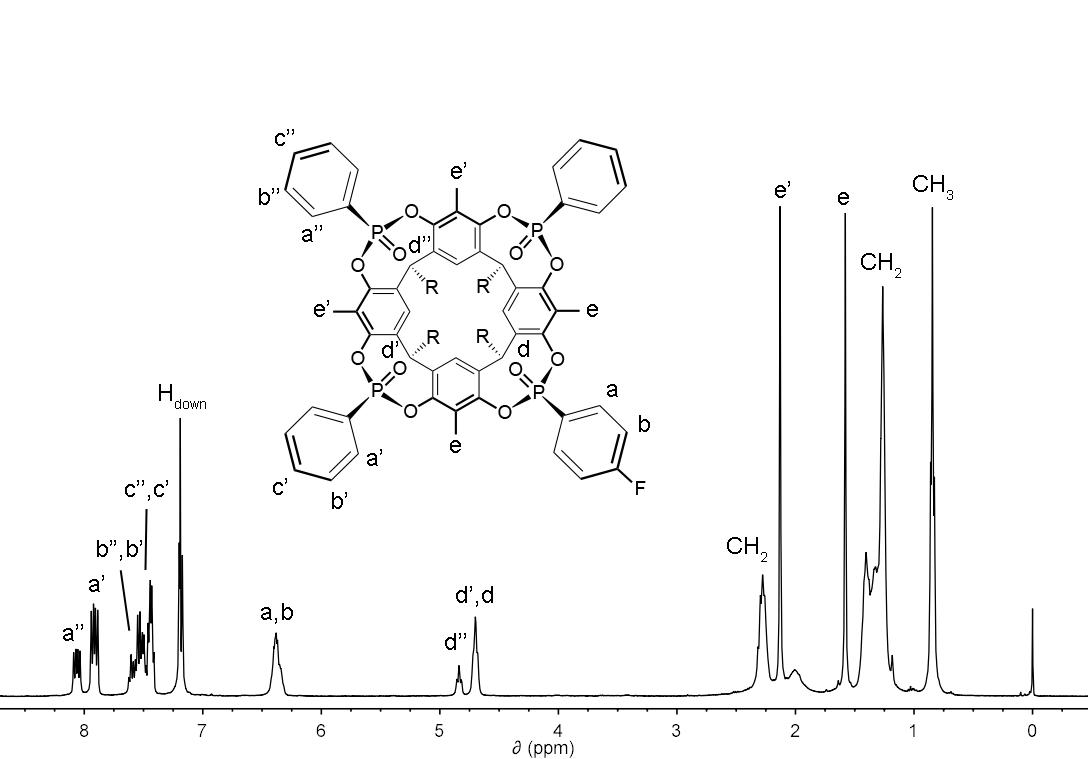


**Figure S4.** ^1^H NMR spectrum (CDCl_3_, 400 MHz, 298 K) of Tiiii [C_6_H_13_, CH_3_, 3Ph + 1PhF_p_] (**2**).


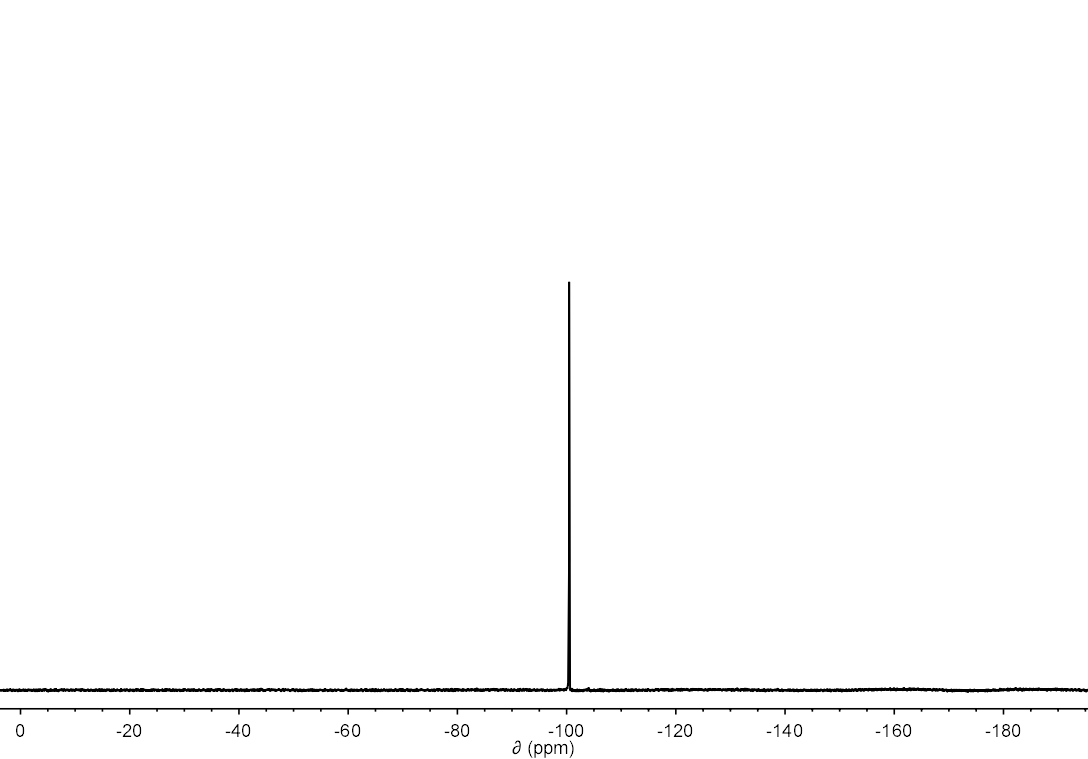


**Figure S5.** ^19^F NMR spectrum (CDCl_3_, 376 MHz, 298 K) of Tiiii [C_6_H_13_, CH_3_, 3Ph + 1PhF_p_] (**2**).


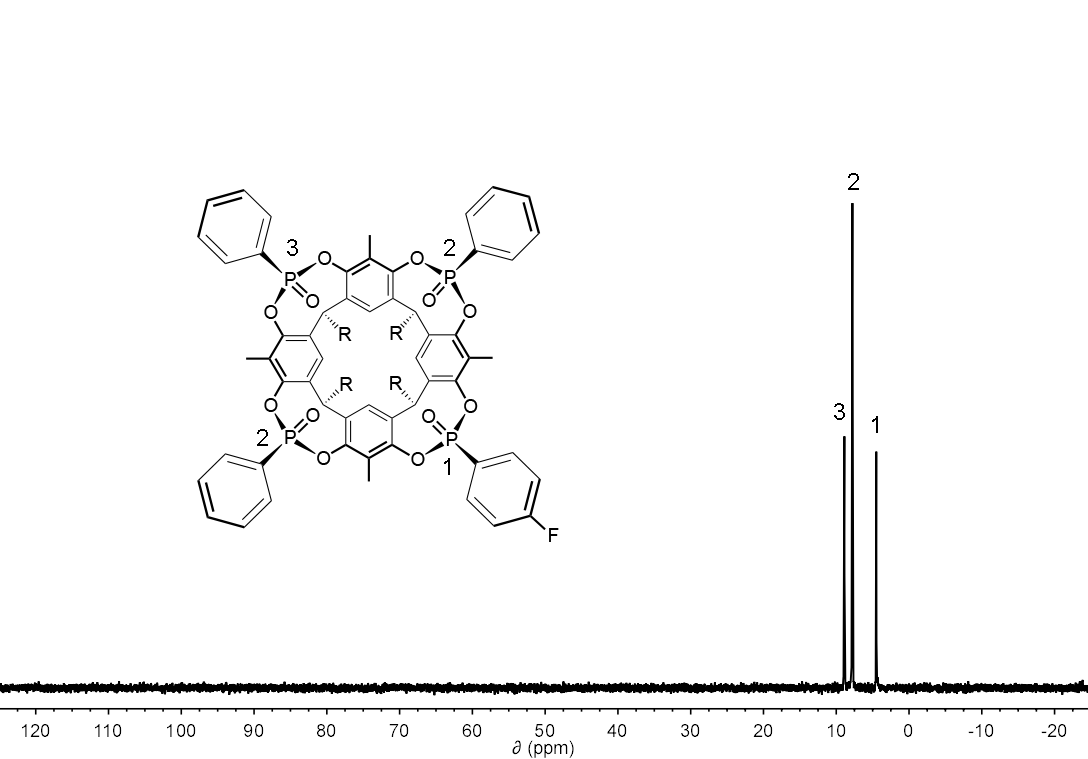


**Figure S6.** ^31^P NMR spectrum (CDCl_3_, 162 MHz, 298 K) of Tiiii [C_6_H_13_, CH_3_, 3Ph + 1PhF_p_] (**2**).

## Mass spectra


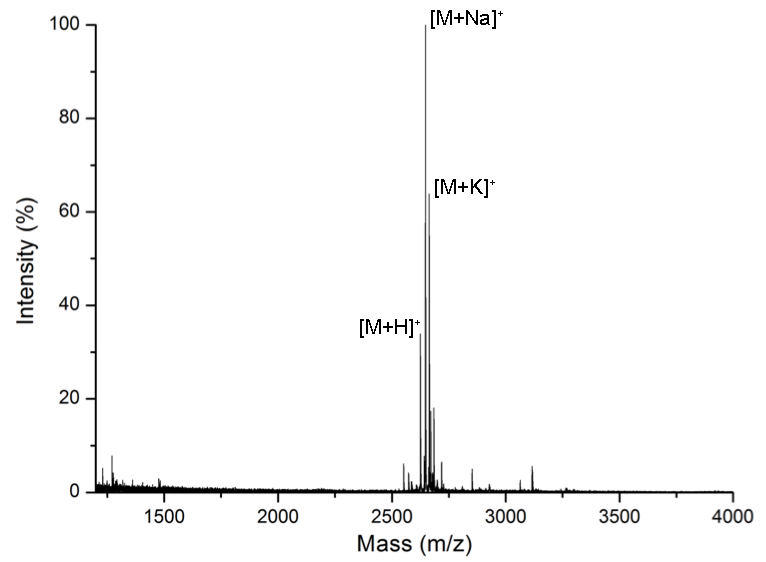


**Figure S7.** High-resolution MALDI-TOF spectrum of Tiiii [(CH_2_)_3_(CF_2_)_7_CF_3_, H, Et] (**1)**


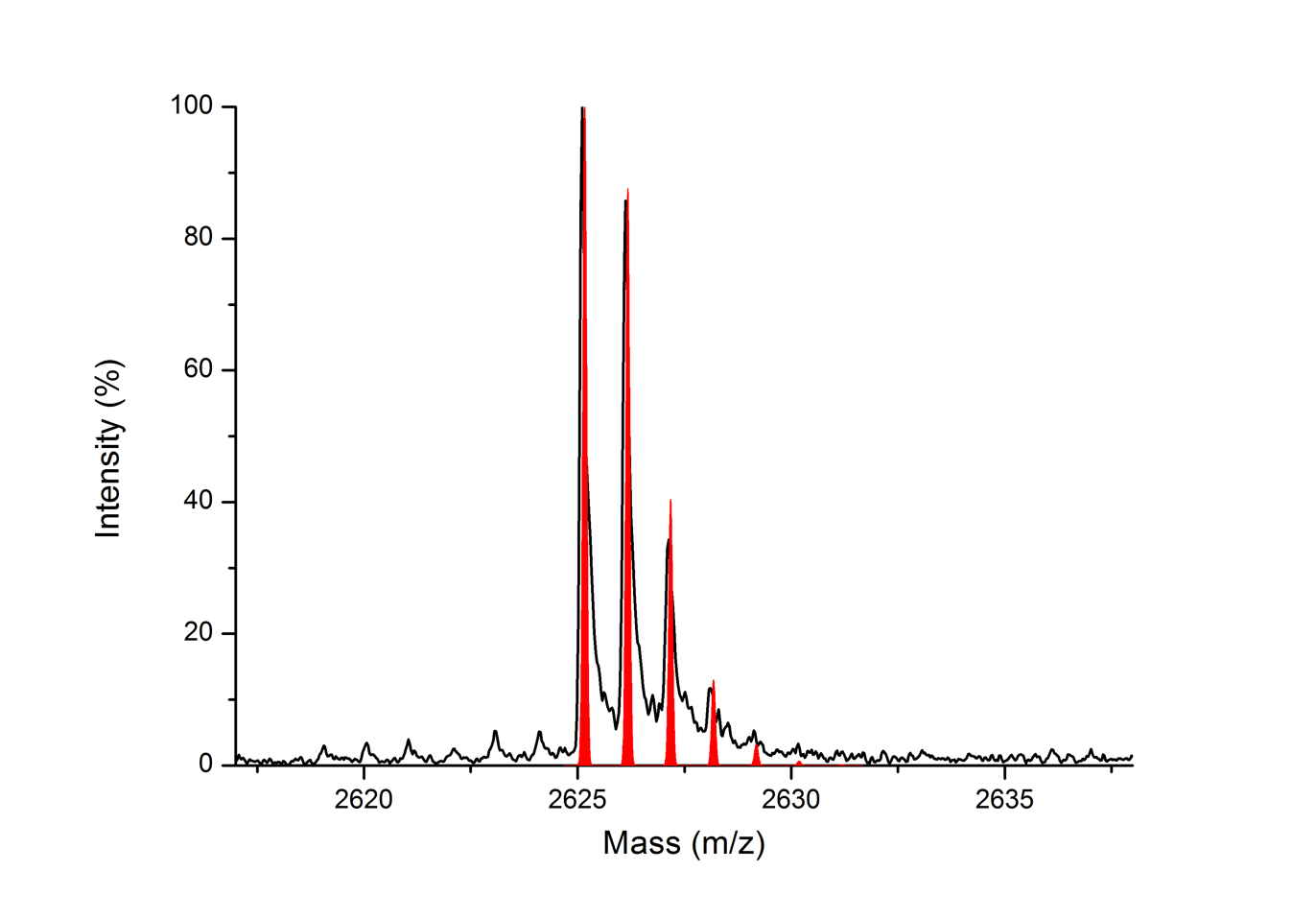


**Figure S8.** Comparison between theoretical (red) and experimental (black) isotopic distribution for [M+H]^+^ ion of Tiiii [(CH_2_)_3_(CF_2_)_7_CF_3_, H, Et] (**1**) as obtained from high-resolution MALDI-TOF analysis.


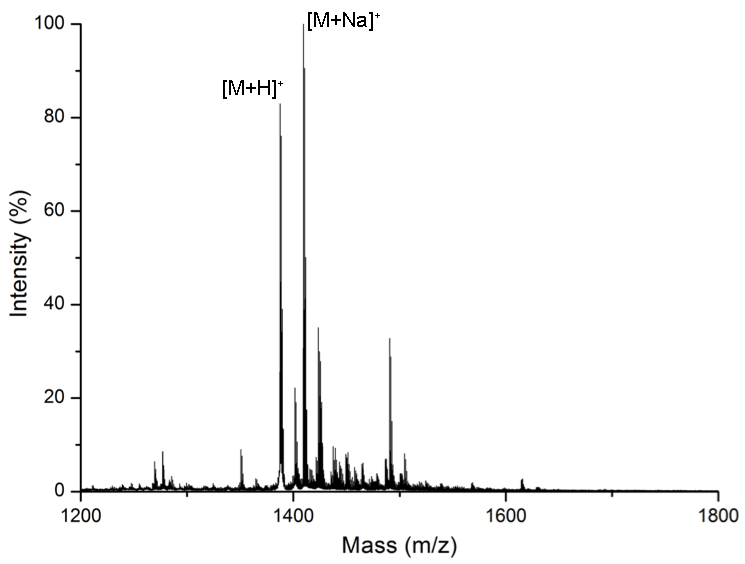


**Figure S9.** High-resolution MALDI-TOF spectrum of Tiiii [C_6_H_13_, CH_3_, 3Ph + 1PhF_p_] (**2**).

**
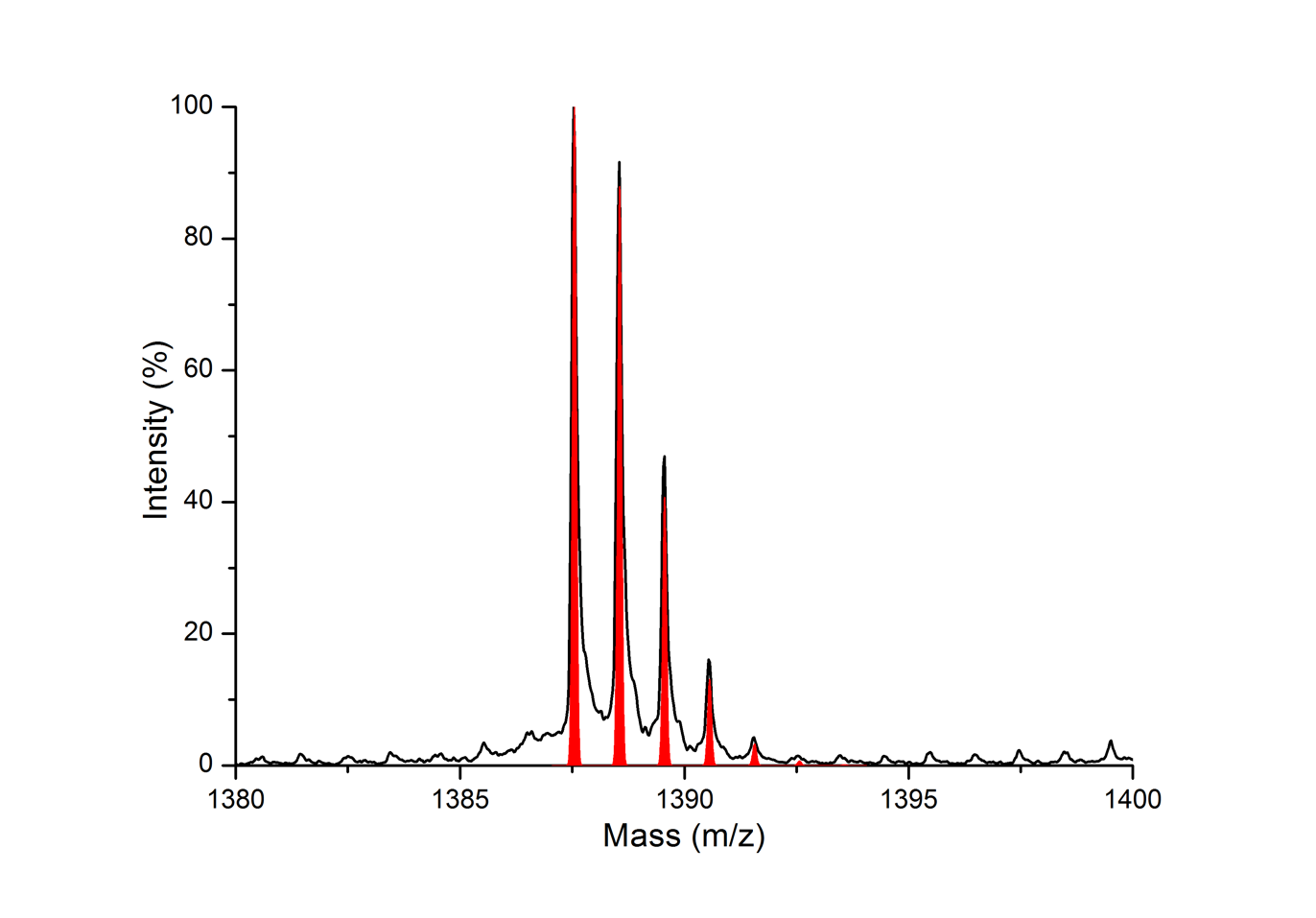
**

**Figure S10.** Comparison between theoretical (red) and experimental (black) isotopic distribution for [M+H]^+^ ion of Tiiii [C_6_H_13_, CH_3_, 3Ph + 1PhF_p_] (**2**) as obtained from high-resolution MALDI-TOF analysis.

# NMR titration experiments


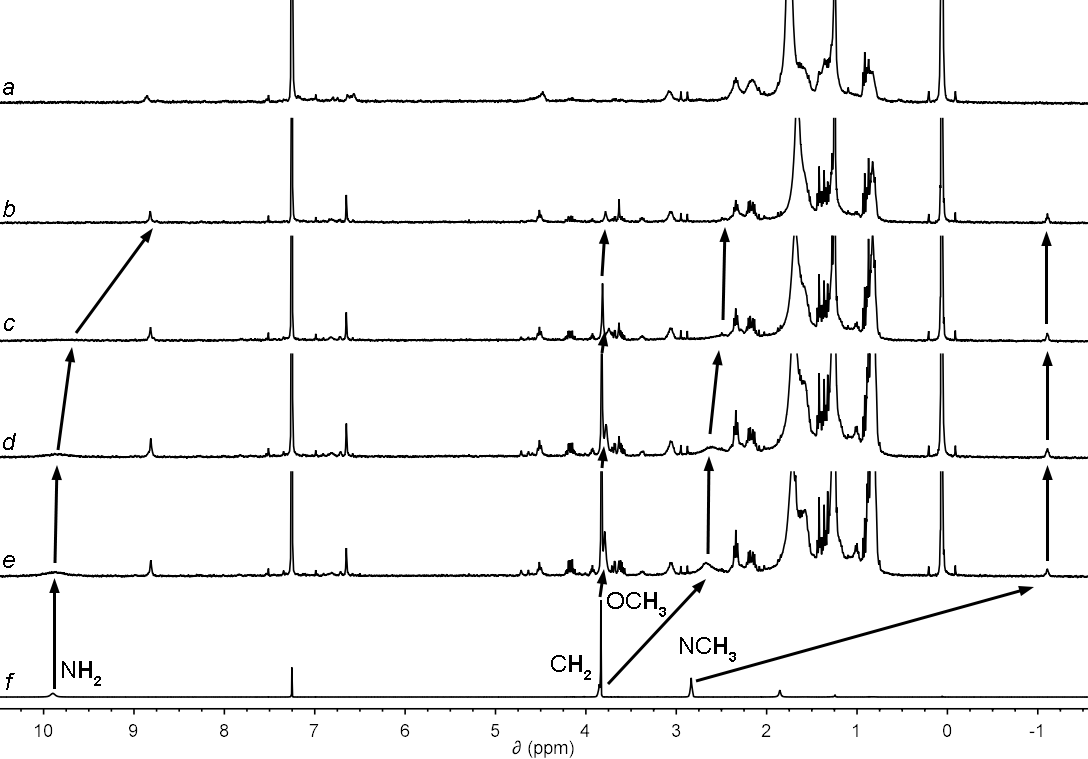


**Figure S11.** ^1^H NMR (400 MHz, CDCl_3_, 298 K) spectra acquired during the titration of **1** with incremental amounts of **G1**. a) free host **1**, [cavitand] = 2 mM; b) **1** + 0.5 eq. of **G1**; c) **1** + 1.0 eq. of **G1**; d) **1** + 1.5 eq. of **G1**; e) **1** + 2.0 eq. of **G1**; f) **G1**.


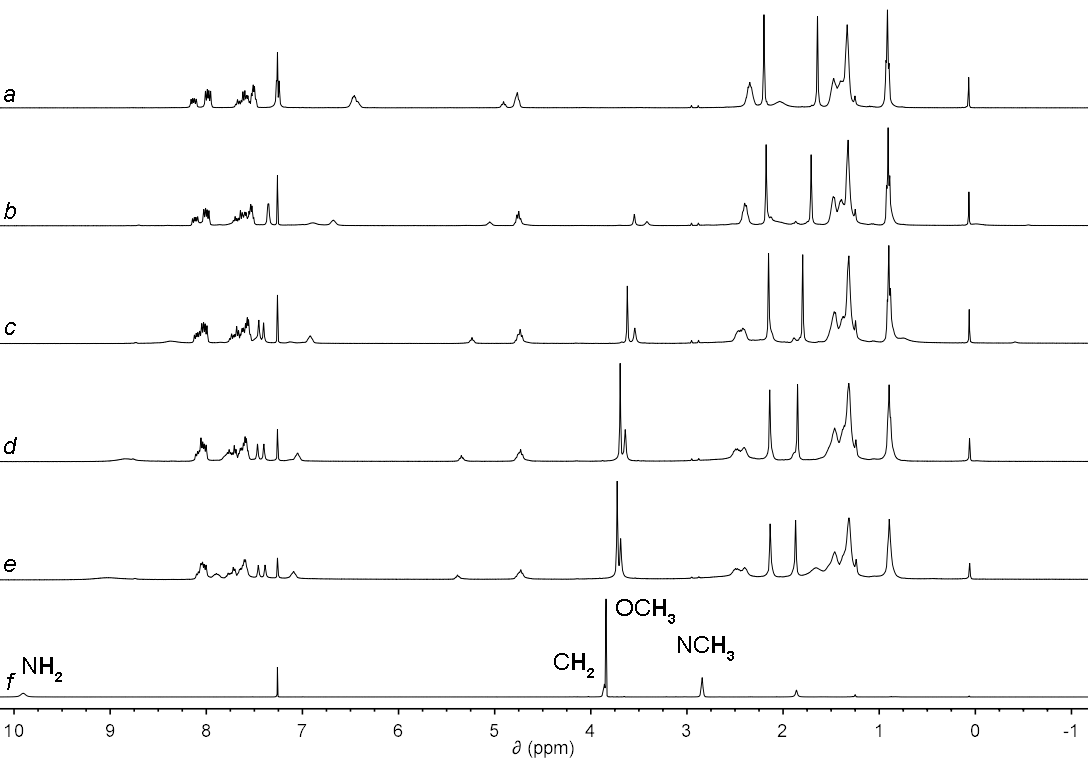


**Figure S12.** ^1^H NMR (400 MHz, CDCl_3_, 298 K) spectra acquired during the titration of **2** with incremental amounts of **G1**. a) free host **2**, [cavitand] = 12 mM; b) **2** + 0.5 eq. of **G1**; c) **2** + 1.0 eq. of **G1**; d) **2** + 1.5 eq. of **G1**; e) **2** + 2.0 eq. of **G1**; f) **G1**.


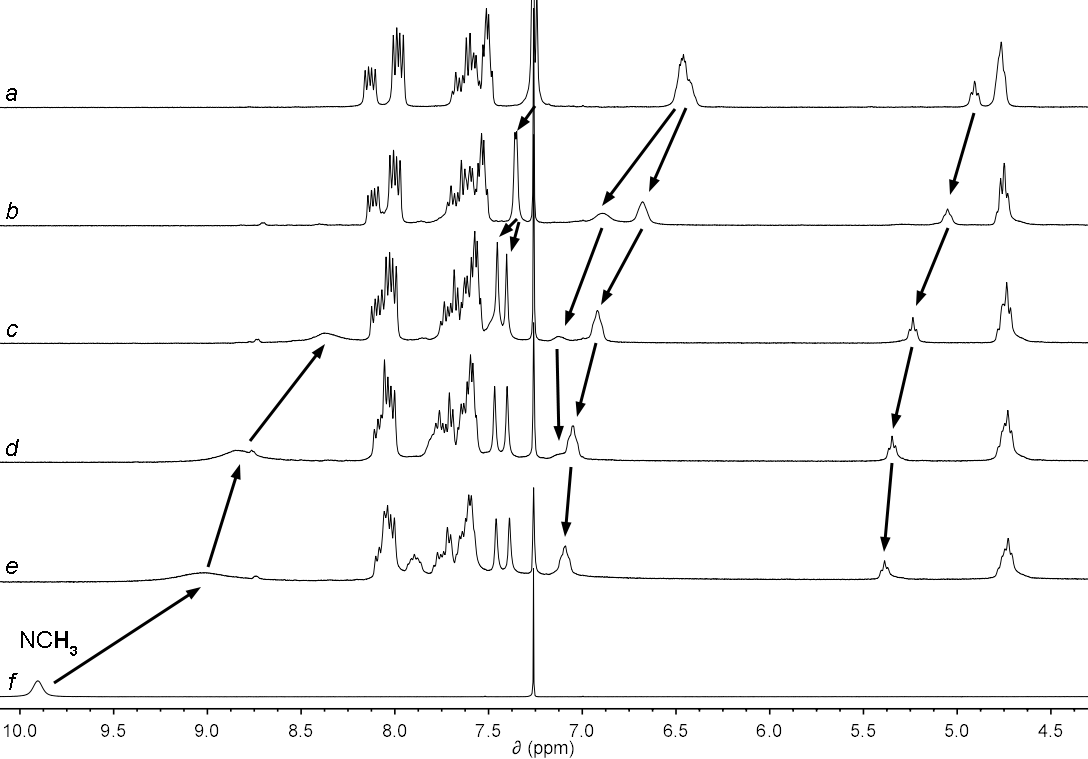


**Figure S13.** Aromatic region of ^1^H NMR (400 MHz, CDCl_3_, 298 K) spectra acquired during the titration of **2** with incremental amounts of **G1**. a) free host **2**, [cavitand] = 12 mM; b) **2** + 0.5 eq. of **G1**; c) **2** + 1.0 eq. of **G1**; d) **2** + 1.5 eq. of **G1**; e) **2** + 2.0 eq. of **G1**; f) **G1**.


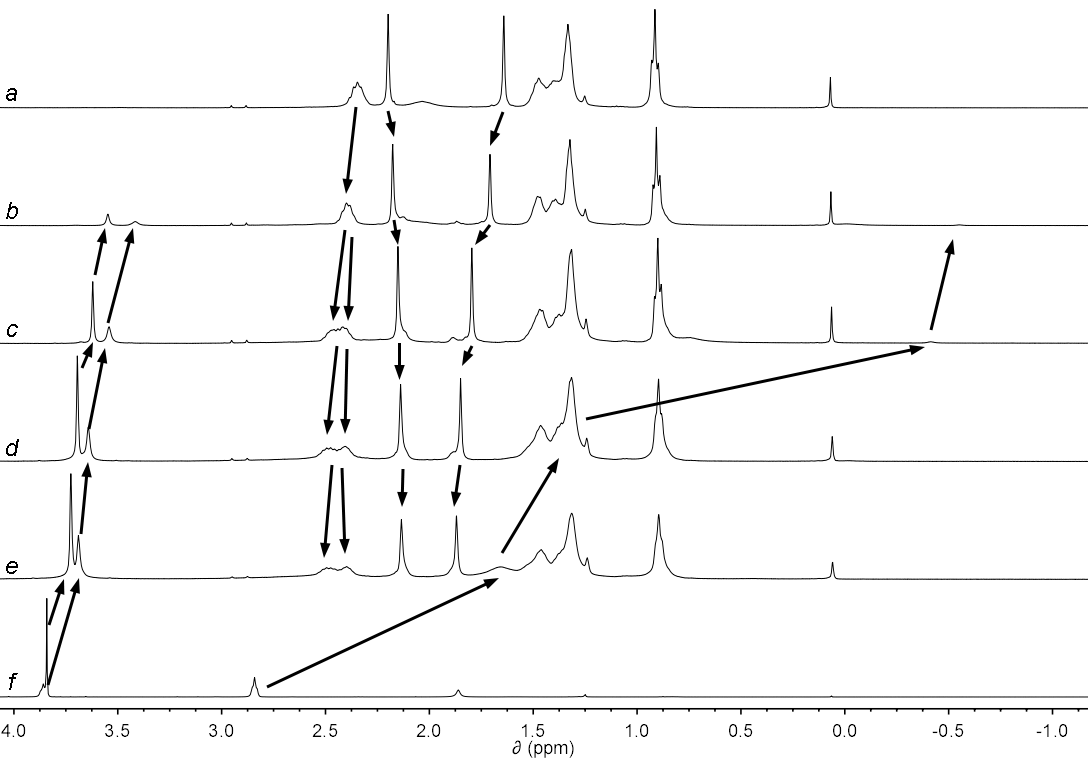


**Figure S14.** Aliphatic region of ^1^H NMR (400 MHz, CDCl_3_, 298 K) spectra acquired during the titration of **2** with incremental amounts of **G1**. a) free host **2**, [cavitand] = 12 mM; b) **2** + 0.5 eq. of **G1**; c) **2** + 1.0 eq. of **G1**; d) **2** + 1.5 eq. of **G1**; e) **2** + 2.0 eq. of **G1**; f) **G1**.


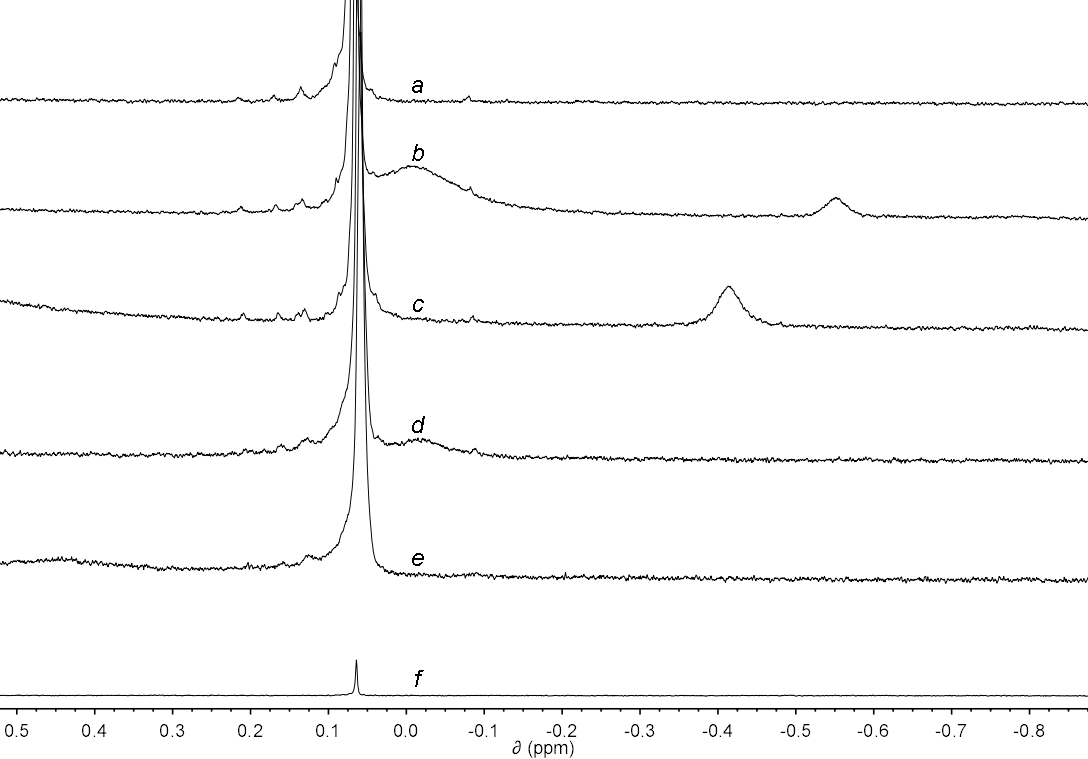


**Figure S15.** Magnification of the negative ppm region of ^1^H NMR (400 MHz, CDCl_3_, 298 K) spectra acquired during the titration of **2** with incremental amounts of **G1**. a) free host **2**, [cavitand] = 12 mM; b) **2** + 0.5 eq. of **G1**; c) **2** + 1.0 eq. of **G1**; d) **2** + 1.5 eq. of **G1**; e) **2** + 2.0 eq. of **G1**; f) **G1**.


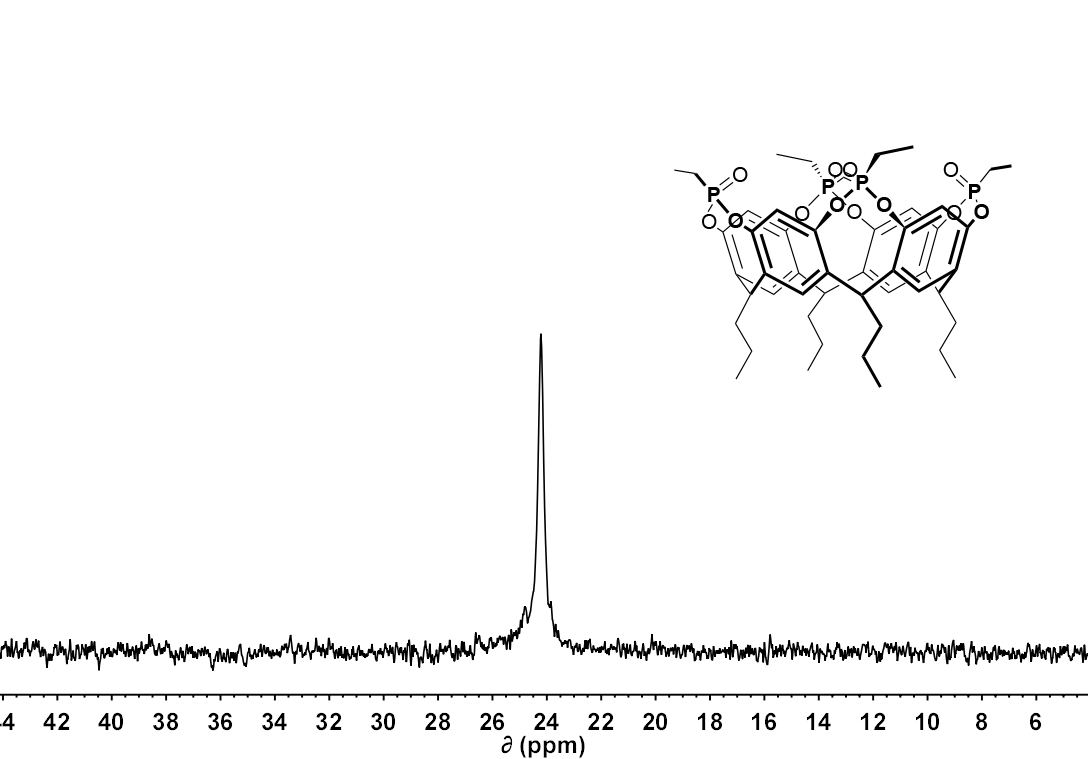


**Figure S16.** ^31^P NMR spectrum (CDCl_3_, 162 MHz, 298 K) of free Tiiii [C_3_H_7_, H, Et].


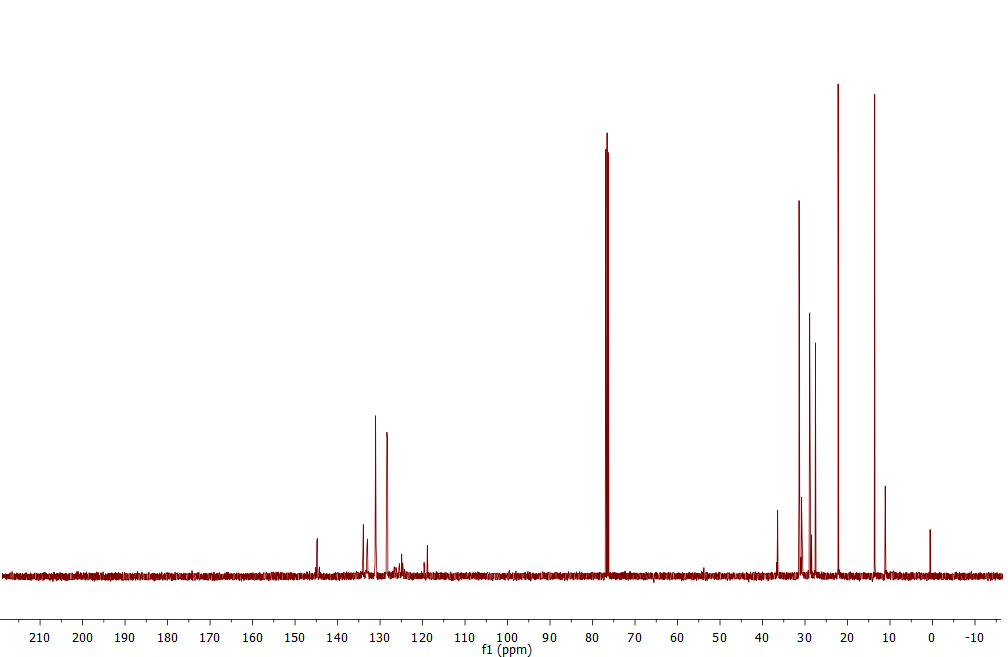


**Figure S17.** ^13^C NMR spectrum (CDCl_3_, 100 MHz, 298 K) of Tiiii [C_6_H_13_, CH_3_, 3Ph + 1PhF_p_] (**2**).
